# The secret life of complement: challenges and opportunities in exploring functions of the complosome in disease

**DOI:** 10.1172/JCI188350

**Published:** 2025-06-16

**Authors:** Tilo Freiwald, Behdad Afzali

**Affiliations:** 1 III. Department of Medicine and; 2 Hamburg Center for Kidney Health, University Medical Center Hamburg-Eppendorf, Hamburg, Germany.; 3 Immunoregulation Section, Kidney Diseases Branch, National Institute of Diabetes and Digestive and Kidney Diseases, NIH, Bethesda, Maryland, USA.

## Abstract

The complement system is a highly conserved and essential immune component with pivotal roles in innate and adaptive immunity. It is increasingly recognized that the complement system has a profound impact on disease. Current complement-targeting therapeutics for clinical use almost exclusively target the complement system in circulation. However, recent discoveries have demonstrated that complement is not only liver derived and plasma operative, but also synthesized and activated inside many cells locally within tissues, performing noncanonical, cell-autonomous intracellular functions, collectively referred to as the complosome. These intracellular complement pathways are distinct from the classical plasma-based system and critical for regulating fundamental cellular processes, including metabolism, gene transcription, autophagy, and the activation and resolution of inflammation. This Review explores the emerging roles of the complosome and current knowledge regarding its relation to human diseases, highlighting evidence across organ systems and disease states, including the kidneys, digestive tract, lungs, heart, CNS, musculoskeletal system, skin, and cancer. We also review current scientific approaches for detecting and functionally investigating the complosome, addressing challenges such as technological limitations and the need for advanced experimental models to delineate its tissue-specific roles. Finally, we discuss central unanswered questions critical for developing innovative therapeutic strategies targeting intracellular complement pathways. These strategies hold potential to modulate disease-specific mechanisms while preserving systemic complement activity.

## Introduction

**Figure 1:**
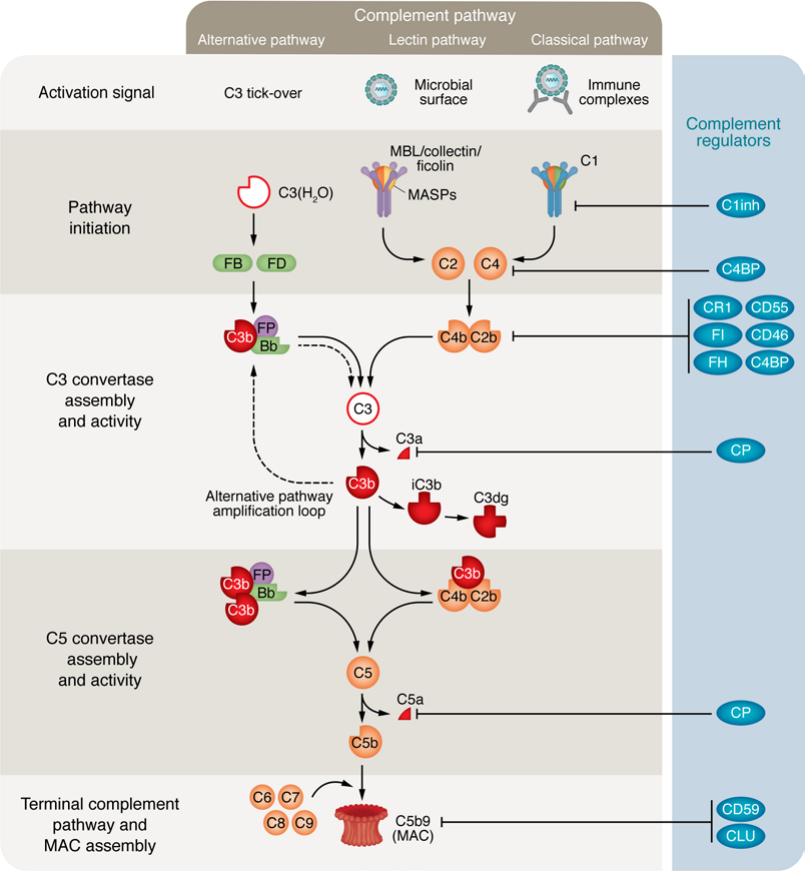
Canonical pathways of complement activation and regulation in circulation. Three principal pathways, the classical, lectin, and alternative, can initiate complement activation upon detection of specific triggers, leading to proteolytic processing of the central components C3 and C5 by their respective multimeric convertases. The generated fragments, alongside formation of the membrane attack complex (MAC), serve as effector molecules (solid red) that clear danger signals through opsonization, complement receptor binding, or direct cell lysis. Multiple regulators (blue) act at several points in the cascade to prevent unintended, prolonged, or excessive activation. Additional serum proteases, including thrombin, which can also activate complement, are not depicted (see Figure 2). MBL mannose-binding lectin; MASPs, mannose-binding lectin serine proteases; FB, factor B; FD, factor D; FP, properdin; C1inh, C1 inhibitor; C4BP, C4b-binding protein; CR1, complement receptor 1; CP, carboxypeptidase; CLU, clusterin.

The complement system is an ancient and evolutionarily conserved component of the immune response, predating the emergence of adaptive immunity by nearly 500 million years. The canonical function of complement as a critical mediator of immunity, essential for recognizing and eliminating pathogen-derived and endogenous danger signals, is indisputable. Briefly, with the exception of complement factor D (CFD), complement components circulate in plasma as inactive precursors, poised for spontaneous or enzymatic activation upon recognition of danger signals. Complement activation’s pathways, classical, alternative, and/or lectin, all converge on a central component, complement factor 3 (C3). Sufficient activation of C3 initiates an amplification loop and triggers downstream effector functions mediated by complement activation fragments or macromolecular complexes (Figure 1). Collectively, these processes confer protection against a wide range of infectious agents and facilitate the clearance of apoptotic and neoplastic cells. These well-established properties of the complement system are discussed in depth elsewhere (1, 2) and will not be the focus of this Review.

Clinical interest in the complement system has grown substantially due to expanding knowledge of its pathological roles; it is now estimated to contribute to the causation and/or perpetuation of up to 400 human diseases (3). As a result, the past two decades have witnessed a surge in the development of therapeutics targeting complement pathways, many of which have successfully entered clinical use (2, 4). Moreover, numerous new potential indications for these therapies and novel agents are on the horizon (2, 5).

While much attention has been focused on the systemic functions of complement, it is clear that complement is also produced within tissues, including the CNS, which is largely insulated from systemic complement (see below). This implies that locally produced complement could act as a surrogate for systemic complement in areas with limited access to the circulation. It is possible, and in some cases evident, that the functions of complement at these sites could mirror its canonical effects, as described over the last 120 years. However, recent studies have also unveiled noncanonical, liver-independent, cell-autonomous complement activities that expand our understanding of complement biology and suggest novel mechanisms of disease pathogenesis and potential therapeutic targets. This renewed interest in the complement system presents additional challenges that need to be addressed. This Review aims to explore these noncanonical functions of the complement system in relation to disease, highlighting the challenges and opportunities they present.

## Local complement and the “complosome”

Traditionally, the complement system was viewed as a liver-derived, plasma-based network of proteins. Although this framework has guided most research on complement activation, extrahepatic production of complement components has been recognized since at least the 1960s. One of the earliest observations came from studies on macrophages, which synthesize multiple elements of the classical and alternative pathways, as well as regulatory proteins (6–16). Such local production can endow macrophages and related cells with the capacity to function independently of circulating complement. Indeed, macrophage-derived C3 can opsonize particles for phagocytosis even in the absence of plasma complement (16). Subsequent findings in lupus-prone mice demonstrated local complement synthesis (*C2*, *C4*, *Cfb*, and *C3* mRNA) in kidney tissues, yet the specific cellular origin and functions of these locally produced components were not fully established (17).

**Figure 2:**
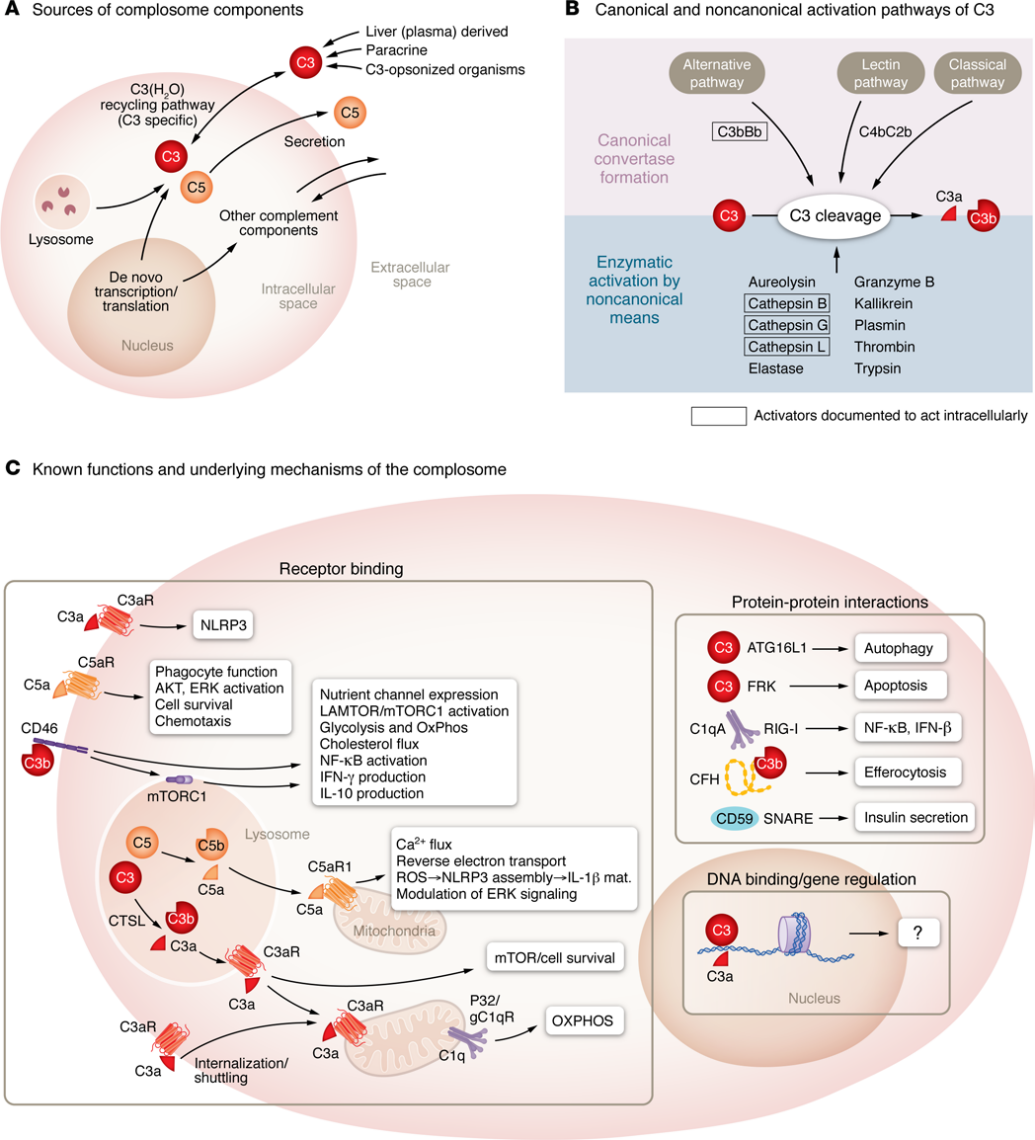
Schematic overview of complosome biology. (**A**) Intracellular complement can originate from de novo gene transcription by cells, uptake from plasma, cointernalization with opsonized pathogens, or from intracellular stores in subcellular organelles. (**B**) C3 activation proceeds via canonical convertases (see Figure 1) or through nonconvertase-dependent proteolysis. Mechanisms shown in boxes have been demonstrated to function intracellularly. By contrast, how C5 becomes activated inside cells remains poorly characterized. (**C**) Complosome functions are illustrated, grouped by protein-protein interactions (top right), receptor-ligand-dependent interactions (left), and DNA-binding mechanisms (bottom right). IL-1β mat., Il-1b maturation.

A critical advancement has emerged recently with the discovery that complement components can be activated inside cells, fulfilling multiple noncanonical, cell-autonomous roles (Figure 2). While the liver remains the predominant source of plasma-circulating complement proteins (18), it is now recognized that many tissues and cell types can synthesize complement locally, especially in regions with limited access to circulating complement, and even activate these components intracellularly. Since this intracellular complement is transcribed from the same genes as its circulating counterpart, specialized cell- or tissue-specific regulatory mechanisms likely govern its expression. The term “complosome” has been adopted to underscore these distinct intracellular pathways. Comprehensive discussions of the complosome can be found elsewhere (19, 20); here, we briefly summarize its key noncanonical functions.

Much of the foundational work on the complosome was conducted in T cells. CD4^+^ T cells harbor intracellular C3 in resting states, partly via uptake of spontaneously hydrolyzed C3(H_2_O) (21) but mainly through de novo transcription (22). Intracellular cathepsin L processes C3 into active fragments (C3a, C3b) that modulate fundamental processes: at baseline, lysosomal C3a supports tonic mTOR activity (22). Upon activation, there is an upregulation of C3 transcription, resulting in increased production of C3a and C3b. C3b is secreted to engage CD46, orchestrating metabolic reprogramming and inflammatory effector functions (22, 23). Additionally, CD46 and TCR signaling activate a cell-intrinsic C5 system, which generates C5a through a yet-to-be-identified protease or convertase. Parallel signaling involving C5a and C5aR1 promotes ROS and inflammasome assembly, promoting inflammation through IL-1β (24). Subsequent signals through CD46 induce antiinflammatory IL-10 and orchestrate shutdown of the inflammatory program (25, 26). Three insights are readily apparent: (a) T cell–derived C3, and possibly C5, can be activated by noncanonical mechanisms; (b) baseline homeostatic functions of the complosome differ markedly from those during activation; and (c) the same component (e.g., C3b) can exert opposing effects at different stages, emphasizing the need for tight spatiotemporal regulation.

Subsequent studies revealed cell-intrinsic expression and, in some cases, biological activity, of numerous complement components, including C1 subcomponents, ficolin-1, C5, CFB and CFD, properdin, C6, C7, C8, C9, C3aR, C5aR1, and C1q receptor (C1qR), as well as various complement regulators (22, 27–30), across immune cell types, suggesting that at least some intracellular complement functions are conserved across different immune cells. Current evidence indicates the complosome influences multiple cellular processes, including gene regulation (23, 31–33), transporter activity (23), cellular metabolism (23, 27, 31, 34, 35), inflammasome activation (24, 27), autophagy (36), and nucleosome phagocytosis (37), among others. However, it is important to note that the mechanisms and functions of the same components may not be identical in every cell type. Notably, myeloid cells assemble a canonical C5 convertase (27), whereas T cells do not (24). Furthermore, intracellular roles are distinct from the capacity of secreted complement to act in *trans*, e.g., endogenously produced C3a and C5a can act on neighboring cells (38, 39), yet cannot fully substitute for intracellular C3 in T cells (40).

These findings highlight the complosome as a critical modulator of basic cellular functions, suggesting that deeper understanding of intracellular complement pathways could provide novel insights into disease mechanisms and open up new opportunities for therapeutic interventions.

## Associations of the complosome with disease

**Figure 3:**
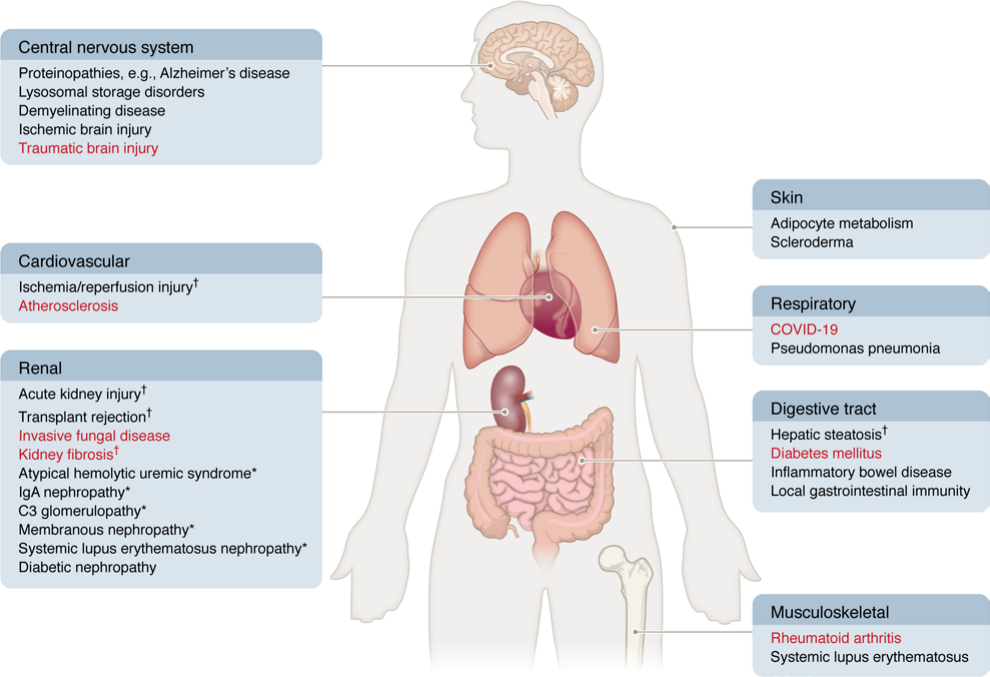
Overview of the complosome in human disease. Diseases are grouped by organ system, with local complement dysregulation listed in black text and complosome-related dysregulation in red text. Daggers denote disorders that rely primarily on model system data. Asterisks denote conditions for which local complement involvement is hypothesized but not definitively proven.

The complosome plays a critical role in regulating cellular physiology in both tissue-intrinsic cells and migrating immune cells that move between different microenvironments. Given this, it is not surprising that abnormalities in the function or regulation of the complosome in either cell type are linked to human diseases. Our understanding of how complosome perturbations result in or modulate disease is currently in its infancy. Many studies have identified dysregulated local expression or upregulation of complement genes in various organs and disease states, speculating that such expression is causatively linked to disease pathophysiology. There is, however, currently a dearth of high-quality in-depth mechanistic studies to definitively prove a causative function. Nevertheless, this is a rapidly expanding area of research, and the following represents the current state of knowledge in this field, organized by organ systems and summarized in Figure 3.

### Kidneys.

As discussed, one of the earliest demonstrations of in situ complement production was in the context of autoimmunity affecting the kidneys of mice (17). Analogous findings in humans were reported by Sacks et al., who isolated mRNA from human kidney biopsies and detected the expression of complement components, including *C3*, in healthy kidneys (41). They noted that *C3* at this site was upregulated in patients with acute kidney injury, including lupus nephritis (41). As before, these studies did not directly attribute complement production to nonimmune cells nor establish a causal link between complement expression and disease manifestations. Subsequent studies have, however, shown that intrinsic kidney cells, such as endothelial, mesangial, and epithelial cells, are capable of producing complement components, particularly following injury. These cells have been shown to synthesize proximal complement components, including C3, C4, and CFB (42–48). The observation that injury drives complement production naturally leads to the hypothesis that inflammatory mediators, such as cytokines, act as upstream signaling intermediaries regulating local complement transcription. Indeed, IL-1 (49, 50), IL-2 (44, 51), IFN-γ (46, 49, 52), and IL-17 (53) have been proposed as positive regulators of local complement production, while TGF-β acts as a negative regulator (54).

Conceptually, the local production of complement in response to kidney injury and inflammatory mediators supports the postulate that its function is to perpetuate local inflammation. Experimental data lend support to this assertion. For example, in rat kidney allografts undergoing ischemia or rejection, peaks in *C3* mRNA expression coincide with elevations of leukocyte-associated cytokines such as IL-2 and IFN-γ (55). Similarly, in murine models of kidney transplantation, wild-type recipients of C3-deficient donor kidneys exhibit delayed acute transplant rejection compared with recipients of C3-sufficient kidneys, possibly due to less aggressive T cell priming in the absence of locally produced C3 (56). However, these studies fall short of providing definitive proof that locally produced complement from nonimmune kidney cells perpetuates inflammation, primarily because other cells within the transplanted kidneys, including passenger immune cells and antigen-presenting cells, are also capable of producing multiple complement components, including C3. Until recently, the lack of mouse models that allow conditional deletion in specific cells, such as C3-floxed strains (see below), has hindered the ability to selectively delete complosome components like C3 specifically in kidney cells. As a result, the functions that these components directly play in kidney cells have not been thoroughly elucidated, nor has the role of kidney cell–derived complement in the response to kidney injury been fully understood.

More recent single-cell and spatial transcriptomic approaches have highlighted a subset of injured kidney tubules that arise de novo following acute kidney injury, for example, after ischemia/reperfusion. These cells have proinflammatory and profibrotic features that are associated with the development of kidney fibrosis (57–60). They express C3, and they are spatially proximate to immune cells that also express C3, especially monocytes (57). Although such injured tubules are associated with fibrosis, the functions of tubule-derived or monocyte-derived C3 in this context are not known. Similarly, podocytes are capable of producing a range of complement components and receptors (61, 62) and are very susceptible to sublytic terminal complement deposition (63), but the role of local production by podocytes has yet-to-be formally dissected. It is plausible that at least a portion of complement deposits observed in complement-associated kidney diseases, such as C3 glomerulopathy, may be produced locally by kidney cells, potentially contributing to the initiation, perpetuation, or scarring processes in these disorders. Because these conditions also respond to extracellular anticomplement therapies, further experimental validation of the contribution of local complement production could help inform the design of next-generation complement-targeting drugs. Nevertheless, recent insights have shed light on the role of the complosome in immune responses to invasive fungal pathogens in the kidneys. Complement component C5 has been identified as a critical factor in protection against invasive candidiasis, which, when unchecked, can lead to acute kidney injury and death in mice. This protective role of C5 is supported by clinical observations showing an increased risk of invasive fungal diseases in patients treated with anti-C5 therapies. Furthermore, individuals carrying *cis*-expression quantitative trait loci (*cis*-eQTL) polymorphisms in the *C5* gene that reduce its expression experience persistent fungemia. Crucially, the protective mechanism of the C5 system against invasive fungal infections involves C5aR1 signaling on myeloid cells in both autocrine and paracrine manners. In this context, myeloid cells within kidney tissues are a key source of C5 and provide C5a for receptor engagement (28). It is noteworthy that the local C5 system also plays a key role in kidney fibrosis (64). The molecular functions of the complosome in fibrosing processes in the kidneys are reviewed in detail in another article in this series (65) and will not be covered here.

### Digestive tract.

Perhaps unsurprisingly, the kidneys are not the only organs capable of synthesizing complement locally. In fact, the repertoire of complosome biology continues to expand as additional studies emerge. Accumulating evidence links multiple roles of the complosome to the digestive tract. There are clear lines of evidence associating extracellular complement dysregulation with hepatic diseases (66, 67) and highlighting an important niche for C3 in hepatic regeneration (68). Importantly, as the largest contributor to circulating C3 (18), the liver synthesizes large quantities of C3, raising the possibility that C3 may also play unappreciated roles within hepatocytes. Indeed, hepatic steatosis is readily apparent in C3-deficient animals, together with impaired very-low-density lipoprotein production due to impaired lipophagy in hepatocytes (69). Here, intracellular C3 interacts with autophagy-related 16-like 1 (ATG16L1) (69, 70), similar to its role in other gastrointestinal cells (36), to regulate autophagosome formation. Interestingly, hyperlipidemia is a relatively common feature of some complement-targeting drugs, such as anti-CFB (71).

Given that diabetes mellitus is a primary driver of hepatic steatosis, the complosome may also indirectly contribute via roles in pancreatic biology. Both C3 and CFB are expressed by pancreatic β cells, especially under cellular stress (72). While systemic CFB and CFD protect against hyperglycemia by generating C3a, an insulin secretagogue (73), intracellularly C3 in β cells protects against IL-1β–induced islet destruction via proapoptotic Fyn-related kinase (FRK) signaling independent of exogenous C3a (72). These studies used β cell–specific C3 knockout mice (RIP-Cre × C3^fl/fl^) (72). Although C3 is also expressed by human β cells (74, 75) and protects against apoptosis, some mechanisms differ from those in mice; for example, those involving upregulated AKT and repressed MAPK (74, 75). How C3 is processed to active forms in β cells, if required, remain unclear. There is, however, evidence that β cells can translate an alternative C3 isoform lacking an N-terminal signal peptide, which is poorly glycosylated, nonsecretory, and opsonizing to intracellular bacteria (76).

Other complement components, including CD59, also aid insulin secretion from β cells. Interestingly, CD59 is a glycosylphosphatidylinositol-anchored (GPI-anchored) membrane protein, yet it is more highly expressed inside β cells (77). Notably, the GPI-anchored form does not appear essential for insulin secretion, whereas global silencing of cellular CD59 significantly impairs secretion by disrupting secretory pathways (77). This mechanism is mediated by interaction between the two isoforms of CD59 and soluble N-ethylmaleimide-sensitive factor attachment protein receptor (SNARE) proteins (78).

Other gastrointestinal cells also produce complement components (79). Local expression of complement in Crohn’s disease was hypothesized in 1990 (80), demonstrated soon after by Northern blotting (45), and C3b was identified in mucosal lesions of inflammatory bowel disease (IBD) (81). Subsequent studies show an enrichment of complement genes among those upregulated in IBD (82) and links to intestinal dysbiosis related to *C4* copy number variations (83). Caco-2 cells express C3, C4, and CFB proteins and upregulate them in response to inflammatory cytokines (84). Colon cells (and mucosal immune cells) express C3 (85–87), C4 (86), and can secrete C3 intraluminally (87). Recent single-cell RNA-Seq and C3-reporter assays indicate stromal cells are the main expressors of C3 in homeostasis, but epithelial cells upregulate C3 during infection (87). In LPS-treated cells, both intact C3 and processed C3 fragments are detected (85), suggesting that intracellular C3 undergoes cleavage. This could be mediated by CFB and CFD (85, 87) or by noncanonical proteases (cathepsins B, L), that can cleave C3 in Caco-2 cells (88). The functional significance of intracellular C3 in intestinal epithelial cells remains unclear, but a role in opsonization of luminal organisms has been proposed (85, 87). Fully C3-deficient mice are susceptible to *Citrobacter rodentium* (87), implicating C3 in protective immunity. Autocrine/paracrine signaling via C3aR may also drive inflammation (85), as shown in bowel ischemia models: epithelial C3 expression is upregulated after injury, and C3-knockout mice are protected (88). Because serum complement depletion does not replicate this effect, locally produced C3 appears pathogenic in this model. Importantly, the key cells mediating pathogenicity here by producing C3, whether primary epithelial cells, immune cells, or both, cannot be distinguished using these full C3-knockout models. In fact, C3 also be protective, as Paneth cell–derived C3 supports proliferation during enteric repair (89).

### Lungs.

Recent studies have illuminated the capacity of respiratory epithelial cells (RECs) to also synthesize complement components. During the COVID-19 pandemic, hyperactivation of the complement system emerged as a critical factor in the pathophysiology of SARS-CoV-2 infection, suggesting an important association between RECs and complement production (90). Infected RECs exhibit pronounced induction of complement gene expression, particularly for genes encoding *C3* and *CFB* (91). The mechanism involves viral sensing that activates type I interferon signaling through the JAK/STAT pathway, directly upregulating complement genes. CFB facilitates the assembly of an inducible, intracellular alternative complement activation convertase, which processes C3 into its active fragments, including C3a (and presumably C3b). Consequently, SARS-CoV-2–infected RECs produce elevated levels of C3a. This overproduction can be attenuated by treating the cells with ruxolitinib, a JAK1/2 inhibitor, or with a cell-permeable inhibitor of CFB (91). Importantly, heightened local production of C3a and C3b by RECs has implications for neighboring immune cells. Immune cells in close proximity to infected RECs respond to the increased C3 activation by upregulating genes associated with C3 fragment receptors, specifically C3aR and CD46 (a receptor for C3b). This response generates a hyperinflammatory signature not observed in circulating immune cells, suggesting a localized amplification of the inflammatory response (91). Beyond modulating immune cell behavior, the elevated C3 production and intracellular C3a within RECs may have direct cell-intrinsic effects. RECs can both synthesize C3 and take up exogenous C3 from external sources (92). Under conditions of cellular stress, such as serum starvation, intracellular C3 has been shown to protect RECs from apoptosis. Conversely, selective deletion of C3 in RECs confers protection against lung injury induced by bacterial pneumonia in vivo. Although the precise mechanisms remain to be fully elucidated, preliminary data suggest that coexpression of CFB meaningfully contributes to these processes (93). In summary, C3 plays an important but complex role in REC survival and injury response. It is plausible to speculate that there exists a therapeutic window for local C3 expression in RECs, where an optimal level of C3 is beneficial for cellular protection but excessive amounts become pathogenic.

### Cardiovascular.

The complement system has been implicated in a variety of cardiovascular diseases, particularly in arterial hypertension and associated end-organ injury (94). This involvement includes not only liver-derived complement components but also CFD, which is predominantly produced by adipose tissue (95). Notably, genome-wide association studies have identified polymorphisms in some complement components, such as *C5*, that are protective against coronary artery disease, although the mechanisms remain unclear (96). Traditionally, cardiovascular outcomes have been linked to systemic complement activity, yet local complement may also be consequential. For instance, C3 deposition in ischemic myocardium appears within three hours of experimental ischemia/reperfusion injury in mice (97), and *C3* deficiency mitigates subsequent necrosis and fibrosis (98). These observations indicate that complement activation contributes to the perpetuation of myocardial damage following ischemia/reperfusion injury. Cytosolic C3 interactions with factors such as cytochrome *c* and procaspase-3 have led to speculation that intracellular complement may protect cardiomyocytes from apoptosis during oxidative stress (99). Further validation via models with cardiomyocyte-specific deletion of complosome components is needed.

Myeloid cells also produce and use complement components in atherosclerosis. For example, *Cfh* deletion in inflammatory macrophages modulates cell-intrinsic C3 activity and mitigates atherosclerosis development by cytoprotective effects in macrophages and lesional efferocytosis (100). Conversely, C5 expression and C5aR1 signaling in myeloid cells promote sterile inflammation, ROS production, *IL1B* gene transcription, and IL-1β protein maturation in cholesterol crystal-rich lesions (27). Nevertheless, despite local complement playing a role in arteriosclerosis, knockout of C3aR1, C5aR1, and C5aR2 in a hypertension mouse model had no apparent effect on hypertension and cardiac injury (101, 102).

### CNS.

The structure of the blood-brain barrier (103) suggests that CNS tissues have limited access to serum components. Therefore, the presence of complement components within the CNS is theoretically more likely to result from local synthesis rather than infiltration from the bloodstream. Indeed, the brain has been recognized as an extrahepatic site of complement synthesis for some time (104–107). Numerous CNS cells, including neurons, astrocytes, microglia, and oligodendrocytes, can produce complement components (106–108). However, it should not be overlooked that complement activation itself can compromise the integrity of the blood-brain barrier (109, 110), thereby permitting serum-derived complement components to enter the CNS.

Complement within the CNS plays critical roles in both normal brain development and the brain’s response to pathological insults. The developmental functions of locally acting complement are well established and include roles in neurogenesis (108), neuronal migration (108, 111), brain remodeling (108, 112), myelination (113), and synaptic transmission (114, 115). These functions have been extensively reviewed elsewhere (108). These physiological functions of complement in the CNS are crucial for understanding its associations with various neurodegenerative diseases. Complement activation has been implicated in proteinopathies such as Huntington’s disease, Alzheimer’s disease, α-synucleinopathies, and age-related macular degeneration (AMD) (108, 116–121). It also plays a role in lysosomal storage disorders like Niemann–Pick disease type C and mucopolysaccharidoses types I and IIIB (108, 116–121). Furthermore, complement is important in demyelinating diseases (122), contributes to protection against CNS infections (107), and participates in the response to ischemic or traumatic brain injury (123–125). The association between the complement system and AMD is noteworthy, as retinal pigment epithelial cells express a range of intracellular complement components, including C3, C3a, C3aR, CR3, and CFB, in response to appropriate stimuli and in association with inflammatory cytokines and inflammasome activation (126). While two complement-targeting drugs (pegcetacoplan and avacincaptad pegol, targeting C3 and C5, respectively) have been approved for the treatment of geographic atrophy in AMD (127, 128), both are extracellular inhibitors. This suggests that complement in AMD may be sourced from the plasma or locally secreted by ocular cells. If the latter is the case, inhibiting local complement *production*, including any intracellular pathways, could potentially offer an even more effective therapeutic approach.

Despite these findings, it remains largely unproven whether the mechanisms of disease in the CNS rely on complement sourced from primary CNS cells, infiltrating immune cells, or leakage from the systemic circulation, and whether complosome components perform intracellular functions in CNS cells similar to their roles elsewhere. For example, single-cell and single-nucleus RNA-sequencing studies have revealed that C1q is highly expressed by glial cells following traumatic brain injury (125) or aging (129). While total knockouts of C1q or blockade using monoclonal antibodies ameliorate clinical features (125), this does not unequivocally prove that locally derived C1q is the driver of disease. Fortunately, the development of cell-specific knockout models, such as microglia-specific C1q ablation, is beginning to conclusively demonstrate the importance of locally synthesized complosome components in the brain. This approach has uncovered a role for microglial C1q in regulating a neurodegenerative profile in these cells (130). Moreover, molecular studies are uncovering intracellular roles for complosome components in CNS cells. For instance, interactions between C1q and neuronal ribonucleoprotein complexes have been shown to regulate neuronal protein translation and brain proteostasis (131). Additionally, C1q interactions with neuronal mitochondria enhance mitochondrial ROS emission and increase the extent of oxidative brain injury following ischemia (132). These findings suggest that intracellular complement components may have important roles in CNS cell function and pathology. Such functional niches might limit the efficacy of anti-C1q antibodies, such as ANX005 (133), unless the neurodegenerative functions of C1q primarily depend on extracellular secretion. Conversely, these therapeutics could help delineate the respective contributions of intracellular versus extracellular C1q to CNS diseases more broadly.

### Musculoskeletal system.

The relative accessibility of cells from inflamed joints and synovial fluids has made studying the role of the complosome in musculoskeletal diseases easier than in many solid organ sites. The relationship between local complement factors and inflammatory arthropathies is evident from the enrichment of complement components and their regulators in the inflamed synovia of patients with rheumatoid arthritis (134). As previously discussed, myeloid and lymphoid cells are important producers of extrahepatic complement. The T cell complosome in this context has well-characterized functions. CD4^+^ T cells migrate into sites of inflammation by diapedesis following the engagement of adhesion molecules expressed on their surface with those on inflamed endothelial cells. For example, lymphocyte function-associated antigen 1 (LFA-1) on T cells binds to intercellular adhesion molecule-1 (ICAM-1, also known as CD54) on endothelial cells. This interaction is crucial because it transduces an AP-1 signal into the nucleus that binds to and transactivates the *C3* gene (40). Thus, the heightened *C3* expression required for the metabolic burst of effector function is regulated by LFA-1 as an upstream factor. The importance of this mechanism in disease is demonstrated by immunodeficiency disorders caused by the lack of LFA-1 expression, such as leukocyte adhesion deficiency type 1 (LAD-1). In these patients, T cells express *C3* poorly and are immunodeficient, failing to generate effective Th1 responses. Remarkably, this deficiency can be rescued by electroporation of *C3* mRNA alone (40). Similarly, elevated *C3* mRNA expression in synovial T cells of patients with rheumatoid arthritis correlates with disease severity (40), and excessive CD46 signaling, the surface receptor engaged by intracellularly processed *C3*, is a pathogenic driver of hyperinflammation at this site (22, 25). Likewise, abnormalities in the regulation of CD46 turnover on CD4^+^ T cells have been proposed as mechanisms underlying hyperinflammation in systemic lupus erythematosus (135).

Mesenchymal cells, such as fibroblasts, are known to express high levels of local complement in joints (136, 137), mirroring their behavior in the gastrointestinal tract. These cells are receiving increasing attention in the literature due to the growing recognition of their important role in the immune system. Recent evidence indicates that they may be responsible for local tissue priming, the retention of immunological memory encoded by epigenetic changes within tissues. Specifically, synovial fibroblasts that are repeatedly stimulated can acquire epigenetic modifications that permit the upregulation of C3 and C3aR, enhancing cellular metabolism and leading to persistent and more aggressive hyperinflammation upon rechallenge at the same synovial site (138).

### Skin.

Noncanonical functions of the complosome are also being explored in other tissues, including the skin. Keratinocytes, skin endothelial cells, and adipocytes are known to produce a range of complement proteins (139–141), and some are sensitive to systemic dysregulation of the complosome (139, 142). In fact, there is an intimate relationship between the complement system and adipose tissue (143), and acquired partial lipodystrophy can be seen in association with systemic hypocomplementemia (144). Deep phenotyping of the complosome using mass cytometry has uncovered specific perturbations of the complosome in circulating T cells from patients with scleroderma, a systemic autoimmune disease affecting the skin (145). More locally, C3a desArg regulates triglyceride synthesis and glucose transport in adipocytes, with dysregulation in this system linked to changes in glucose tolerance (146, 147). It is important to note, however, that some data reporting metabolic effects of C3a desArg have faced challenges in replication (148), as discussed elsewhere (149). Additionally, recent work has demonstrated that the adipsin/C3a/C3aR1 axis regulates thermogenesis in beige/brown adipose tissues, revealing sexually dimorphic effects on adaptive thermogenesis and cold tolerance (150). These findings suggest that the complosome may play a substantial role in skin physiology and metabolic processes. Undoubtedly, in-depth studies examining the specific functions of the complosome in the skin will be forthcoming.

### Cancer.

The role of the complosome in cancer is very complex and contradictory. This is because there are multiple sources of complement in the tumor microenvironment: tumorous cells themselves, complement expressed by mesenchymal cells (e.g., cancer-associated fibroblasts), tumor-infiltrating immune cells, and complement made available from the circulation, which is likely to be more efficient given the angiogenesis associated with cancers. For these reasons, the literature describes both positive and negative effects of the complosome on cancer progression, and these are comprehensively reviewed elsewhere (151–153) and in other reviews in this series (154, 155).

## Current challenges in understanding and exploiting the complosome

Despite remarkable advancements, many unanswered questions remain regarding the nature and function of the complosome, its effect on human diseases, and how it can be therapeutically manipulated. In the following sections, we discuss current scientific approaches to investigating the complosome and highlight key unknowns that require elucidation.

## Approaches for detecting and functionally exploring the complosome

There are multiple methods for detecting the expression of complosome components. These range from inexpensive, readily available options to sophisticated methods that require considerable expertise in use and interpretation. These methods are summarized in Table 1, along with the relative strengths and weaknesses of each. Briefly, at the transcript level, qRT-PCR offers a straightforward but low-throughput method; bulk RNA-Seq provides more comprehensive data but lacks cellular context. Single-cell or single-nucleus RNA-Seq addresses this by capturing individual cell transcriptomes, albeit at high cost and with patchy data for low-abundance transcripts. Spatial transcriptomics adds geographic coordinates in tissue sections yet remains expensive and technically demanding. Protein-level assessments are critical, given that mRNA may not correlate well with protein activity (156, 157). Clinical tests can assess complement functionality in serum, but they do not distinguish local or intracellular activities. Western blotting can detect specific cleavage fragments, though antibodies may not be able to differentiate subcellular or processed forms. Mass spectrometry, including imaging modalities, offers detailed analysis of fragments and posttranslational modifications but typically requires specialized expertise. Proximity ligation assays can detect convertase assembly in situ. Functional studies, including cell- or tissue-specific knockout mice, siRNA-mediated knockdown, and reporter mice, allow direct testing of the roles of complosome components. Finally, identifying specific posttranslational modifications may enable distinguishing between intracellularly synthesized and circulating complement (76) as complement can be imported into cells from the microenvironment (21) or via complement-coated pathogens (158).

## Potential therapeutic strategies

With increasing awareness of the roles played by the complosome in human diseases, greater attention is being directed toward the therapeutic targeting of complement in local spaces. In some cases, the accessibility of discrete anatomical sites makes this readily achievable; for example, injection of complement inhibitors directly into the eye for the management of geographic AMD (4) or into the oral cavity for the management of periodontal disease (159). Adeno-associated virus targeting can also deliver therapeutic molecules to regulate complement activity, and specificity can be imparted by incorporating promoters that are active only in specific tissues. The success of adeno-associated virus–mediated delivery of therapeutic cargo is highly tissue dependent but is continually improving (160, 161). Many alternative approaches for cargo delivery are available, such as bispecific molecules (162), but a major challenge remains the anatomical location of many complosome components. Similarly, there is considerable effort to develop complement-targeting siRNA (163, 164) and cell-specific conjugates for targeted delivery to specific tissues and organs (165). Such strategies hold promise for more precisely modulating the complosome and potentially addressing intracellular complement activity. The intracellular location of many complosome components presents a challenge for direct targeting, as cell-permeable molecules are required. These molecules are challenging to develop, can have off-target or toxic effects, and may act systemically on both intracellular and extracellular complement. Alternatively, rational target selection for efficient modulation of the complosome can be achieved by gaining a better understanding of key regulatory nodes, such as the upstream signals that induce local complement transcription, the processes that generate active fragments, the location and structure of complosome components, and the precise function of each component in a given tissue or cell. Much of this information remains currently unknown and is the subject of further research.

## Key unanswered questions

### Location and trafficking.

Our understanding of the complosome remains in its infancy, with many unanswered questions. A key area is the subcellular localization of complosome components, which can provide clues about their function and interactions. For example, C3aR and C5aR1 have been found on “unexpected” organelles such as lysosomes and mitochondria, suggesting specialized roles that may be cell-type or context specific. Distinguishing the molecular and spatial differences between locally derived and systemic complement will aid targeted drug development, whether by modifying upstream signals, manipulating intracellular processing, or targeting activation fragments. Some intracellular interactions, such as C3 with cytochrome C or procaspase 2 and C1q with ribonucleoprotein complexes (99, 131), suggest complement proteins reside in the cytosol rather than being restricted to membrane-limited organelles. Understanding how complement proteins reach distinct compartments is another key to uncovering new therapeutic strategies.

### Structure.

If intracellular complement functions similarly to its extracellular counterpart, subcellular environments may influence structural integrity. For example, C3 in β cells can be translated from an alternative start codon and lacks a signal peptide, allowing cytosolic localization (76). The reducing conditions of the cytosol are generally inhospitable to proteins that require disulfide bonds, raising questions about the stability of C3 and function in this compartment. While in vitro studies suggest C3 is quite resistant to reducing conditions (20, 74), it remains unknown whether intracellular complement requires fidelity to its canonical structure and/or if it undergoes cell-specific posttranslational modifications or conformations. Defining intracellular complement structures could inform the development of targeted inhibitors that selectively modulate complosome activity without affecting circulating complement.

### Transcription.

The regulation of complement gene transcription within cells offers a potential target for therapy. For example, type I interferons drive *C3* and *CFB* transcription in RECs, an effect reversible with JAK inhibitors such as ruxolitinib (91). In CD4^+^ T cells, LFA-1 transactivates *C3* via the AP-1 complex (40). The fact that different cell types use distinct transcriptional cues to regulate the same complement genes suggests opportunities for cell-specific therapeutic interventions. Further research is needed to delineate the regulatory networks that govern local and cell-specific complement production.

### Activation and function.

Complement production does not necessarily translate to activity, as C3 and C5 require cleavage for canonical functionality. While convertases mediate this process classically, noncanonical enzymes such as cathepsins and thrombin can also activate complement (22, 166), but their relative dominance in different tissues is unclear. Human protein and RNA atlases are helping identify key tissue-specific activators. Once activated, some complement components require interacting receptors to exert effects. The tissue-specific distribution of receptors, such as C3aR1, C5aR1, and C5aR2, remains debated*,* partly due to conflicting RNA-Seq data. Additionally, receptor-independent signaling exists, as seen in C3 interactions with ATG16L1 in autophagy (69, 70). Coexpression patterns that hint at additional potential activators are currently under assessment.

The distinct roles of locally produced versus systemic complement remain poorly defined. C3 allotype analysis in kidney transplant patients suggests locally produced C3 can contribute up to 16% of total plasma C3 (167), and in inflammatory environments, local C3 could dominate. This may be particularly relevant in immune-privileged organs where systemic complement access is limited.

### Local niche.

Tissue susceptibility to complement activation varies widely. For example, hepatocytes produce large quantities of complement proteins, yet complement-driven liver diseases are rare, despite activation in inflammatory liver conditions (66, 67). In contrast, kidneys are highly vulnerable to complement overactivation, leading to severe damage and potential organ failure (168–170). The nephron’s ultrastructure may contribute to complement entrapment, and evidence suggests complement plays opposing roles in hepatic regeneration (68) versus kidney fibrosis (65). Understanding these differential susceptibilities could reveal new organ-specific therapeutic targets.

### Conservation of function.

Finally, murine models provide valuable insights, but species differences must be accounted for. For example, the murine ortholog of CD46 does not perform the same functions as its human counterpart, which regulates C3b and signals in T cells (23, 25, 26). Findings from mice should therefore be validated in human systems. Organoids offer a promising contemporary alternative for studying local complement effects in human tissues, bridging the gap between in vivo and in vitro research (171).

## Summary and future directions

Recent exciting developments in understanding the complement system have begun to unravel the critical importance of its functional localization, including its roles within the intracellular space. The “complosome” represents a rapidly expanding field that offers profound insights into the basic molecular biology of cells and mechanisms of disease, unveiling novel avenues for therapeutic intervention. Advancements in this area are progressing in tandem with technological developments that enable high-throughput, multi-omic interrogation of cellular and tissue environments. As a nascent field, many unanswered questions remain, the resolution of which will hopefully pave the way for the design of next-generation therapeutic strategies targeting the complement system within this anatomical location.

## Figures and Tables

**Table 1:**
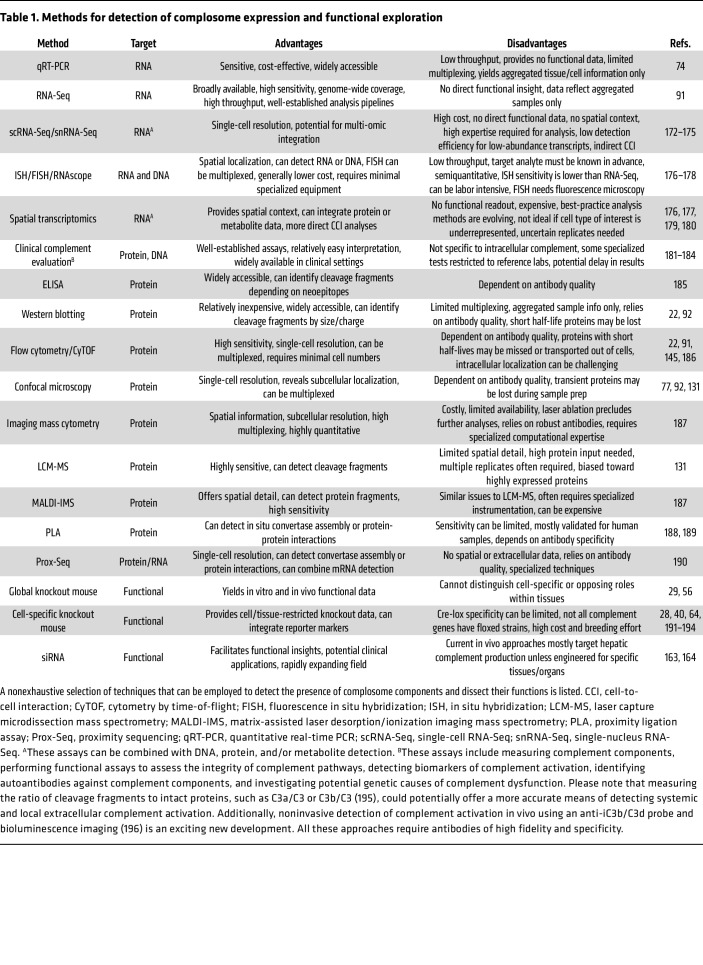
Methods for detection of complosome expression and functional exploration

## Version 1 06/16/2025

Electronic publication
